# Supplementary material for: Deletion of wfs1 Impairs Oligodendrocyte Precursor Cells Dorsal Distribution and Myelination Through the wfs1-hmgcs1 Axis in Zebrafish
Source: Biology (Basel). 2026 Aug 16;15(16):1402. doi: 10.3390/biology15161402 (PMC13510151; doi:10.3390/biology15161402)
Supplement: Supplementary file 1 [file biology-15-01402-s001.zip › File S1-Transcriptome sequencing data and differentially expressed genes (DEGs) in wfs1-- zebrafish at 5 dpf/2.KEGG/wfs1KOvsWT/GSEA/WT/NUCLEOTIDE_EXCISION_REPAIR/NUCLEOTIDE_EXCISION_REPAIR(DRE03420).html]

Details for gene set NUCLEOTIDE\_EXCISION\_REPAIR(DRE03420)[GSEA]

|  || Dataset | deg.sample |
| Phenotype | sample.cls |
| Upregulated in class | WT |
| GeneSet | NUCLEOTIDE\_EXCISION\_REPAIR(DRE03420) |
| Enrichment Score (ES) | -0.21104477 |
| Normalized Enrichment Score (NES) | -0.87736726 |
| Nominal p-value | 0.6394422 |
| FDR q-value | 0.7883262 |
| FWER p-Value | 1.0 |
Table: GSEA Results Summary

  

Fig 1: Enrichment plot: NUCLEOTIDE\_EXCISION\_REPAIR(DRE03420)      
 Profile of the Running ES Score & Positions of GeneSet Members on the Rank Ordered List

  

| PROBE | DESCRIPTION (from dataset) | GENE SYMBOL | GENE\_TITLE | RANK IN GENE LIST | RANK METRIC SCORE | RUNNING ES | CORE ENRICHMENT || 1 | 393774 | pole3 |  |  | 941 | 2.179 | 0.0342 | No |
| 2 | 556995\_1 | lig1 |  |  | 956 | 2.157 | 0.0969 | No |
| 3 | 100006257 | cetn2 |  |  | 1540 | 1.633 | 0.1264 | No |
| 4 | 449811\_1 | ercc8 |  |  | 1629 | 1.583 | 0.1700 | No |
| 5 | 402864 | uvssa |  |  | 2903 | 1.058 | 0.1610 | No |
| 6 | 450080 | mnat1 |  |  | 4196 | 0.794 | 0.1436 | No |
| 7 | 556456\_1 | pold1 |  |  | 4702 | 0.718 | 0.1488 | No |
| 8 | 100037336 | rfc1 |  |  | 5563 | 0.631 | 0.1402 | No |
| 9 | 541386 | xpc |  |  | 6070 | 0.580 | 0.1413 | No |
| 10 | 324323 | ercc3 |  |  | 7211 | 0.473 | 0.1193 | No |
| 11 | 449992 | polr2i |  |  | 8143 | 0.416 | 0.1022 | No |
| 12 | 259256\_1 | rfc3 |  |  | 8868 | 0.377 | 0.0905 | No |
| 13 | 100003720 | polr2m |  |  | 8893 | 0.375 | 0.1007 | No |
| 14 | 100149268 | polr2b |  |  | 9501 | 0.334 | 0.0914 | No |
| 15 | 259256 | rfc3 |  |  | 9590 | 0.327 | 0.0982 | No |
| 16 | 560477 | ercc6 |  |  | 10392 | 0.279 | 0.0812 | No |
| 17 | 65226 | rpa2 |  |  | 10674 | 0.262 | 0.0801 | No |
| 18 | 192320 | pole2 |  |  | 10741 | 0.259 | 0.0856 | No |
| 19 | 406435 | rfc4 |  |  | 10835 | 0.253 | 0.0901 | No |
| 20 | 692256 | pold3 |  |  | 11051 | 0.241 | 0.0904 | No |
| 21 | 445284 | rad23aa |  |  | 12484 | 0.165 | 0.0502 | No |
| 22 | 100330570 | polr2ea |  |  | 12494 | 0.165 | 0.0547 | No |
| 23 | 553347 | polr2a |  |  | 13422 | 0.119 | 0.0291 | No |
| 24 | 327322 | ercc4 |  |  | 13473 | 0.117 | 0.0309 | No |
| 25 | 100003720\_1 | polr2m |  |  | 13653 | 0.109 | 0.0285 | No |
| 26 | 556456 | pold1 |  |  | 13726 | 0.106 | 0.0293 | No |
| 27 | 796456 | pold2 |  |  | 13771 | 0.103 | 0.0310 | No |
| 28 | 796803 | polr2k |  |  | 14176 | 0.084 | 0.0207 | No |
| 29 | 556995 | lig1 |  |  | 14356 | 0.076 | 0.0173 | No |
| 30 | 554099 | polr2j |  |  | 15654 | 0.014 | -0.0230 | No |
| 31 | 445170 | polr2eb |  |  | 16081 | -0.005 | -0.0363 | No |
| 32 | 393196 | rpa3 |  |  | 16744 | -0.024 | -0.0564 | No |
| 33 | 571834 | si:dkey-28b4.7 |  |  | 16805 | -0.027 | -0.0575 | No |
| 34 | 445385 | rfc5 |  |  | 16927 | -0.032 | -0.0603 | No |
| 35 | 324088 | polr2gl |  |  | 17039 | -0.034 | -0.0628 | No |
| 36 | 550263 | polr2h |  |  | 17204 | -0.041 | -0.0668 | No |
| 37 | 553610 | pole4 |  |  | 17453 | -0.052 | -0.0731 | No |
| 38 | 327491 | rpa1 |  |  | 17594 | -0.058 | -0.0758 | No |
| 39 | 562839 | rad23ab |  |  | 17912 | -0.072 | -0.0836 | No |
| 40 | 553405 | pole |  |  | 18294 | -0.089 | -0.0930 | No |
| 41 | 447857 | gtf2h1 |  |  | 18522 | -0.100 | -0.0972 | No |
| 42 | 751713\_1 | gtf2h5 |  |  | 18626 | -0.105 | -0.0974 | No |
| 43 | 30678 | pcna |  |  | 18983 | -0.121 | -0.1050 | No |
| 44 | 334669 | polr2c |  |  | 19911 | -0.162 | -0.1294 | No |
| 45 | 393599 | ddb1 |  |  | 20019 | -0.167 | -0.1279 | No |
| 46 | 393536 | rad23b |  |  | 20084 | -0.170 | -0.1249 | No |
| 47 | 334839 | gtf2h4 |  |  | 20549 | -0.191 | -0.1339 | No |
| 48 | 436588 | polr2d |  |  | 21291 | -0.230 | -0.1505 | No |
| 49 | 503748 | rfc2 |  |  | 22293 | -0.284 | -0.1736 | No |
| 50 | 560313 | cul4b |  |  | 22929 | -0.322 | -0.1842 | No |
| 51 | 393900 | ercc2 |  |  | 23690 | -0.372 | -0.1972 | No |
| 52 | 394002 | cul4a |  |  | 23739 | -0.376 | -0.1877 | No |
| 53 | 100001425 | ercc1 |  |  | 24484 | -0.419 | -0.1988 | Yes |
| 54 | 449846 | rbx1 |  |  | 24797 | -0.438 | -0.1958 | Yes |
| 55 | 553705 | polr2l |  |  | 25124 | -0.455 | -0.1927 | Yes |
| 56 | 323239 | gtf2h2 |  |  | 25307 | -0.467 | -0.1847 | Yes |
| 57 | 541502 | ercc5 |  |  | 25801 | -0.511 | -0.1853 | Yes |
| 58 | 393443 | xpa |  |  | 25847 | -0.514 | -0.1716 | Yes |
| 59 | 436837\_1 | gtf2h3 |  |  | 25966 | -0.526 | -0.1599 | Yes |
| 60 | 100004133 | ddb2 |  |  | 26394 | -0.566 | -0.1568 | Yes |
| 61 | 449811 | ercc8 |  |  | 26532 | -0.582 | -0.1440 | Yes |
| 62 | 541325 | ccnh |  |  | 26657 | -0.593 | -0.1305 | Yes |
| 63 | 436817\_1 | polr2f |  |  | 26668 | -0.595 | -0.1135 | Yes |
| 64 | 553705\_1 | polr2l |  |  | 27280 | -0.647 | -0.1137 | Yes |
| 65 | 405897 | cdk7 |  |  | 27781 | -0.713 | -0.1085 | Yes |
| 66 | 553405\_1 | pole |  |  | 30393 | -1.444 | -0.1483 | Yes |
| 67 | 556995\_2 | lig1 |  |  | 30840 | -1.799 | -0.1097 | Yes |
| 68 | 393774\_2 | pole3 |  |  | 31836 | -4.872 | 0.0017 | Yes |
Table: GSEA details [plain text format]

  

Fig 2: NUCLEOTIDE\_EXCISION\_REPAIR(DRE03420)      
 Blue-Pink O' Gram in the Space of the Analyzed GeneSet

  

Fig 3: NUCLEOTIDE\_EXCISION\_REPAIR(DRE03420): Random ES distribution      
 Gene set null distribution of ES for **NUCLEOTIDE\_EXCISION\_REPAIR(DRE03420)**
